# Editorial

**Published:** 1863-08

**Authors:** 


### Editorial.

Medical Colleges in Chicago.—The two regular medical colleges in this city have issued their respective announcements for the coming annual courses of instruction.

That of the Rush Medical College, indicates no important alterations from the preceding year, either in the course of instruction or in the members of the Faculty. The term will commence on the 7th of October, and continue, as usual, *sixteen weeks.* For clinical instruction, the Faculty seems to rely almost wholly on the college dispensary, or rather on a simple college clinic twice a week. The announcement alludes to an arrangement for admitting the students into the Marine Hospital, but it does not state how often, or by whom they are to be instructed when there. A few weeks since, we saw the Annual Catalogue and Announcement of the University of St. Mary's-of-the-Lake, a well-known catholic college in this city, in which the Faculty of Rush Medical College are represented as the *Medical Department* of that University. The same claim to a Medical Department is contained in the advertisement of that University, in the daily papers of this city. In the Annual Announcement of the Rush Medical College, now before us, there is no allusion to the subject. Has the Rush Medical College and the Catholic University really become united, or is the statement in the circular of the last-named institution a mere advertising dodge on the part of the Rush College? The same circular of the University of St. Mary's, stated that candidates for the degree of M.D., in that institution, must have studied medicine, *at least*, *one year!* Yes, *one* whole year of study to make a "Doctor of Medicine!" The year is, doubtless, intended to include *sixteen weeks* of lectures in the, so called, Medical Department. Verily, this affords a *short*, if not a royal road to medical honors, or, more properly, *titles*. A full explanation of the condition and prospects of the Chicago Medical College, (the Medical Department of Lind University,) we give in the July number of this Journal, and hence will not repeat it here.

The new college building, there described, is rapidly approaching completion. The Mercy Hospital has become fully installed in its new and pleasant quarters, and its wards are more full than they have been at any previous time during the last two years. They will afford an ample field for true clinical instruction, in reference to all the more important diseases met with in medical and surgical practice. The students of the Chicago Medical College, in addition to the usual college clinics twice a week, will receive regular clinical instruction in the wards of the Hospital, four mornings in the week, throughout the whole college term. From the Announcement of the Rush Medical College, we learn that the General Introductory Lecture, at the opening of their next Annual Course, will be delivered by E. INGALLS, M.D., Professor of Materia Medica and Medical Jurisprudence, on the evening of the 7th of October.

The General Introductory Lecture, at the opening of the next term in the Chicago Medical College, will be delivered by N. S. Davis, M.D., Professor of Practical Medicine, &c., on the evening of October 12th.

---

Army Surgeons.—We are informed that all vacancies in the Medical Department of the Army, so far as relates to the Illinois Volunteers, have been filled; and, consequently, that there is no necessity for further applications for examination and appointments at present.

---

### TO THE MEDICAL PROFESSION OF ILLINOIS.

The undersigned was, at the last meeting of the Illinois State Medical Society, appointed as Chairman of the Committee on Drugs and Medicines.

All members of the Medical Profession in the State of Illinois, are hereby requested to report the result of their observations in regard to any new remedies, or new application of those long known, to the Chairman, at Quincy, Dr. R. G. Laughlin, of Hayworth, or Dr. F. R. Payne, of Marshall.

Respectfully, F. K. BAILEY.

*Chairman Committee on Drugs and Medicines.*

Quincy General Hospital, August, 1863.

---

Illinois State Medical Society.—The Special Committee on *Diseases of the Eye*, appointed at the last meeting of this Society, earnestly requests all members of the Association, and of the profession generally, to aid in accomplishing the objects for which the Committee was appointed.

Although the Committee has previously reported upon the causes of conjunctival inflammations as they prevail at the West, it is desirable to collect more facts in reference to the causes and prevention of this disease.

The Committee would suggest, that all members, who have observed epidemics of conjunctivitis, contribute such facts as may demonstrate to what extent the disease is influenced by dryness of the atmosphere, season of the year, dust from trees and plants, want of care as regards food and exposure, malaria, etc.

The history of interesting cases of any form of ophthalmic disease is urgently solicited.

It is important that all communications be forwarded to the address of the subscriber, previous to April 1, 1864.

E. L. HOLMES,
Box 2175. *Committee on Diseases of the Eye.*

---

ARTIFICIAL PETRIFACTION OF ANIMAL TISSUE.—In one of his interesting European letters to the *American Medical Times*, descriptive of the principal hospital and other medical institutions of Florence, Prof. Charles A. Lee thus notices a very important discovery, a knowledge of which appears to have been lost through the cold indifference to new ideas and narrow prejudice against progress which so generally prevails, to the great disadvantage of science and the injury of those noble pioneers of truth who promote its advancement.

"In the museum of this school, also, are the celebrated preparations of Legato, who died about 30 years ago. This celebrated anatomist discovered a mode of changing all animal tissues into stone, without changing their form or color in the slightest degree, and even preserving the natural flexibility of the ligiments, tendons, and joints, etc. Here is a tablet, perhaps a foot and a-half square, inlaid with splendid mosaics in ornamental figures, consisting entirely of the various textures of the body converted into stone, hard and smooth as polished marble. For example, a portion of liver, lung, spleen, skin, kidney, penis, uterus, cartilage, muscle, brain, nerve, spinal cord, membrane, eye, bone, etc., etc., all retaining their natural color, and readily recognized by the anatomist. This celebrated genius did not meet with that encouragement which he expected and deserved for his most important discovery, the Government entirely ignoring his valuable services, and the secret accordingly perished with him. Among the animals converted into stone I noticed the rat, cat, spider, fishes, etc, all looking perfectly natural."—*Druggists' Circular.*

---

GARIBALDI PROBE.—Dr. E. L. Duer, of the Sixteenth U. S. General Hospital at Philadilphia, proposes a very simple substitute for the porcelain-headed probe of M. Nelaton, known by surgeons as the *Garibaldi probe.* He suggests a white or opalescent glass rod, having successfully made use of this for several months. He says, in a note to the *Philadelphia Reporter*, "A rod of the requisite thickness and color may be simply and readily prepared by first rounding the end a little, by holding it in the flame of a spirit-lamp, and then rasping it off with emory paper."—*Cincinnati Lancet and Observer.*

---

MICHIGAN STATE UNIVERSITY.—At the recent annual meeting of the trustees of this institution, Dr. Tappan was removed from the Presidency and the Professorship of Sacred Rhetoric, and Dr. E. O. Haven elected to fill the vacancy. The election was made *viva voce* and unanimous. It is understood that hereby is removed the noxious influence which has year after year persisted in the effort to foist a chair of Homœopathy on the medical department of this University.—*Cincinnati Lancet and Observer.*

---

LARGE SCROTAL TUMOR.—Dr. Cleveland, of Malabar, has reported the removal of a large scrotal tumor, from a native of the Laccadive Islands, the weight being 78 pounds. The man was a strumose subject, and died on the ninth day after the operation, from an attack of diarrhœa.

